# Microsurgical training on non-living models: proposal for basic training

**DOI:** 10.1515/iss-2024-0039

**Published:** 2025-06-03

**Authors:** Mara Franza, Giovanni Maria D’Antonio, Anna Barbara Di Stefano, Emanuele Cammarata, Giovanni Zabbia, Francesca Toia

**Affiliations:** Department of Precision Medicine in Medical, Surgical and Critical Care - Plastic and Reconstructive Surgery, 18998 University of Palermo, Palermo, Italy; BIOPLAST-Laboratory of BIOlogy and Regenerative Medicine-PLASTic Surgery, Plastic and Reconstructive Surgery, Department of Precision Medicine in Medical, Surgical and Critical Care – Plastic and Reconstructive Surgery, University of Palermo, Palermo, Italy

**Keywords:** microsurgery, microsurgical training, basic course, non-living models

## Abstract

**Objectives:**

Microsurgery is an essential skill for plastic surgeons and a basic microsurgical course should be integrated into all plastic surgery residents’ training programs, as mastering this technique cannot be reached only through observation and requires regular practice. Despite living models better simulate reality, non-living models have been shown to be valid tools for basic/intermediate microsurgical training. While many single preclinical exercises are described in the literature, there is lack of proposals for a complete basic program on non-living models. The aim of this study is to propose a basic microsurgical program entirely based on non-living models and to evaluate its feasibility with a cross-sectional study that analyses the outcomes of its application via the “Queen Mary University London microsurgery global rating scale” scoring system.

**Methods:**

Nine different exercises for microsurgical training on non-living models were chosen based on a literature review. A basic step-by-step microsurgical training program was built. The program was proposed to the Plastic Surgery residents of our unit through a five-day training, during 2020–2023. No participants ‘selection was made. All participants were examined with a practical test before and after the proposed program. The results of pre- and post-training test were collected and analysed.

**Results:**

Each trainee was able to complete a posterior-wall-first technique end-to-end anastomosis on the chicken thigh femoral artery with no major mistakes at the end of the program. Moreover, the data analysis revealed a statistically significant improvement of each evaluated skill after the training.

**Conclusions:**

The proposed basic program is easy to organize and allows the trainee to develop the basic microsurgical skills needed to move to advanced training on living animals or on guided clinical practice.

## Introduction

Microsurgery is a crucial part of a Plastic Surgeon’s surgical background, as it allows complex reconstructions to be managed. Dedicated microsurgical training is essential to acquire and develop the needed microsurgical skills and a basic microsurgical course should be integrated into all Plastic Surgeon residency training programs. Aims of basic training include getting confident with the operative microscope, microsurgical instruments, and threads, developing hand-eye coordination under magnification, improving fine tissue dissection and learning vascular and nerve suture techniques.

Basic microsurgical training is usually set up with the first phases on non-living models and the advanced ones on living animal models, mostly on living rats [1]. The growing interest in animal rights and in 3R principles (replace, reduction and refinement) [2] has led to the development of many alternatives on which to perform not only basic but also advanced microsurgical training, such as food [3], synthetic materials [4], 3D virtual reality [5], 6] and non-living animal models [7], [8], [9], [10], [11]. Several non-living models are described in literature: synthetic materials, such as vinyl or latex gloves [12], food [3], and non-living animal models, such as chicken [10], 13], pig [11], 14], and even some small human samples [15], 16]. While exercises on synthetic materials are designed to learn and improve the basic gestures, including instrument and needle handling, wrist movement and manual finesse, and often can be interchanged to rehearse the same skills (e.g. exercises on gauze or on vinyl gloves), exercises on non-living animals aim at developing more advanced skills and provide a more realistic simulation experience due to tissue consistency [17]. The chicken thigh is the most used non-living animal model for microsurgical training due to its clear neurovascular bundle and structural similarities to human tissue [7]. It allows the trainee to practice with tissue dissection, vessels preparation for anastomosis, adventectomy, both with vascular and nerve suture and provides vessels ranging in diameters from 2 mm to 0.5 mm or less, making the training more challenging when requested [8]. Furthermore, the chicken thigh can be used for practicing on flap raising [18]. Several studies have already supported the use of chicken thigh for enhancing microsurgical techniques and have validated it as an effective model for microsurgical training [10], 13], 19].

Non-living models have globally many advantages: they are cheap, easily accessible and allow practice starting from the first steps of training on microsurgical instrument handling and hand-to-eye coordination to the more challenging exercises as those on the chicken leg even for supermicrosurgical training [20]. However, they have some limitations. The training on non-living models is easier compared to that on living models: they don’t require extensive dissection for neuro-vascular bundle exposure, there is no tissue bleeding or pumping blood flow to manage during the anastomosis, and loss of blood after clamps removal; they do not offer the experience of vasospasm or thrombus formation after vessels manipulation and the training is stressless because there is no correlation between any surgical act and animal death. Despite these drawbacks, they allow the trainee to efficiently learn the basis of microsurgery and prepare them for the most advanced phases of training on living models.

We believe that an established basic microsurgical program, even if utilizing non-living models, could facilitate widespread learning of microsurgery for its suitability. Its feasibility, due to easily accessible materials, low costs, and the absence of ethical concerns, can also help surgical residents and young surgeons maintain or enhance their existing skills.

The aim of this study was to propose a basic microsurgical program based entirely on non-living models and to evaluate its feasibility through its application to plastic surgery residents. The proposed program was not intended to be an alternative training to that on living animals, since the latter certainly allows the trainee to develop more advanced microsurgical skills.

## Materials and methods

A review of the literature was performed [17], based on which different exercises on non-living models were selected. A 25 h basic course was designed, according to national guidelines for basic training of the Italian Society for Microsurgery (SIM) [1]. Nine different exercises on non-living models were chosen. The exercises were proposed following with increasing difficulty. The first 7 h were spent on non-animal models, while the following 18 h were spent on the chicken thigh model. The program was followed by 30 Plastic Surgery Residents at our Institution, during 2020–2023, in a five-day course. All residents were included in the study. No participant’s selection was made. The residents had various levels of prior experience in microsurgery. Each trainee was supervised by a consultant (trainee/supervisor ratio 1:1).

Each trainee performed at least 55 anastomoses in line with the minimum standards set by the International Microsurgery Simulation Society (IMSS) consensus [21] (15 on non-animal models, and 40 on non-living animal models). At least 25 end-to-end and 10 end-to-side anastomoses were performed on the chicken femoral artery and vein, and at least 10 neurorrhaphies were performed on the chicken femoral nerve. Each trainee was evaluated either before and after the proposed training program by 3 experienced senior microsurgeons, while making an end-to-end anastomosis on chicken femoral artery The “Queen Mary University London microsurgery global rating scale” [22] was used for the evaluation. The final score attributed to each item on the “Queen Mary University London microsurgery global rating scale” was unanimously assigned after the collegial evaluation carried out by the three judges. Data on achieved scores were collected and analysed. Descriptive statistics were carried out through mean and range for quantitative variables. Paired samples *t*-test was used to compare the pre- and post-training scores for each item and the total score of the “Queen Mary University London microsurgery global rating scale”. Statistical significance was set at p<0.05. We followed the institutional guidelines approved by the local Ethical Committee of our University Hospital, stating that no ethical approval is required for studies that do not involve humans or living animals. The study was conducted according to STROBE guidelines for cross-sectional studies [23].

### Program and exercises

Our program includes preliminary exercises performed without the aid of the microscope, to improve manual dexterity and coordination. They include exercises with sushi chopsticks, on gauze, and with needles with eyelets. Microsurgical exercises, which can only be performed with the use of the microscope are exercises on the plate, on the grape, on the silicone tube, on the rubber mouse, on the noodles and finally on the chicken thigh. The exact sequence of exercises and the skill rehearsed are presented in Table 1.

**Table 1:** Detailed sequence and visual representation of the nine exercises included in the microsurgical training program.

Exercise	Time	Equipement	Execution	Skill rehearse	Example
Sushi chopsticks	1 h	Container, two sushi sticks, items to be moved (beans, grains of rice, lentils)	Moving the objects one by one from the outside to the inside of the container	Develop fine manual skills and dexterity	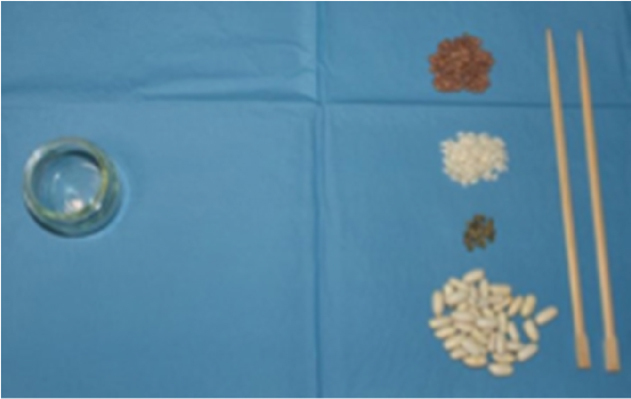
Stiches on gauze	1 h	Nylon 6-0 suture thread, white gauze, two patches, curved microsurgical needle holder, microsurgical forceps	Put stiches on a white gauze fixed with two patches to avoid any movement during the exercise	Develop fine manual skills and dexterity	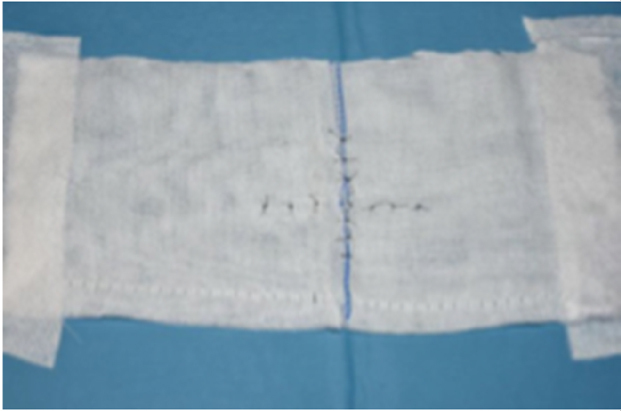
Needle exercise with buttonhole	1 h	A polystyrene panel, 24 needles with eyelets arranged in pairs, a 8-0 suture thread, curved microsurgical needle holder, microsurgical forceps	First inserting the distal end of the needles into the polystyrene panel covered with a green cloth. Then passing the microsurgical suture needle through the eyelets of the 12 pairs of needles arranged in a circle, so as to form a “double clock”	Develop fine manual skills and dexterity	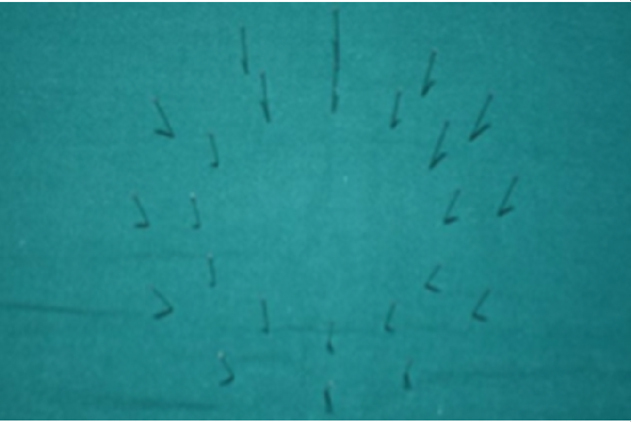
Grape berry dissection	1 h	A grape berry, two pins, polystyrene panel, scalpels, microsurgical forceps, microsurgical scissors	Fix the grape berry with pins to the polystyrene panel. Draw a star on the grape skin. Then start the fine dissection of the star-shaped skin grape with microsurgical scissors under magnification	Improve fine dissection and manual finesse under magnification	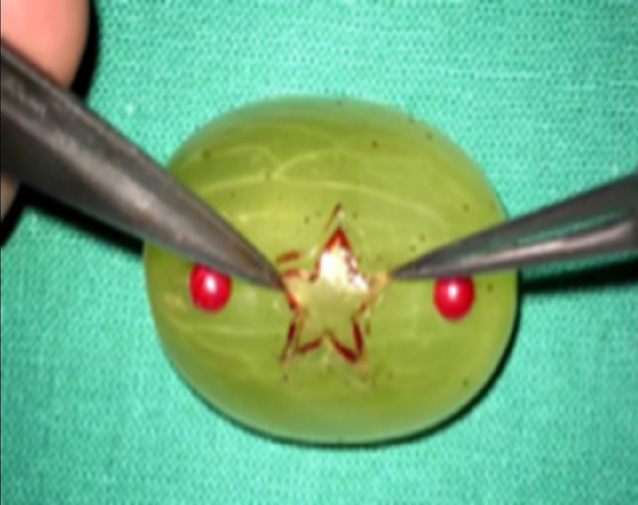
Plate exercise	1 h	A plastic cup, no-sterile latex glove (size S), some stones, nylon 6-0 suture thread, scalpels, curved microsurgical needle holder, microsurgical forceps	Secure a surgical glove on the surface of the glass with an elastic band, after placing stones inside to make the glass stable. After cutting the plate with a scalpel, the sutures are carried out with the aid of the microscope	Develop fine manual skills and dexterity	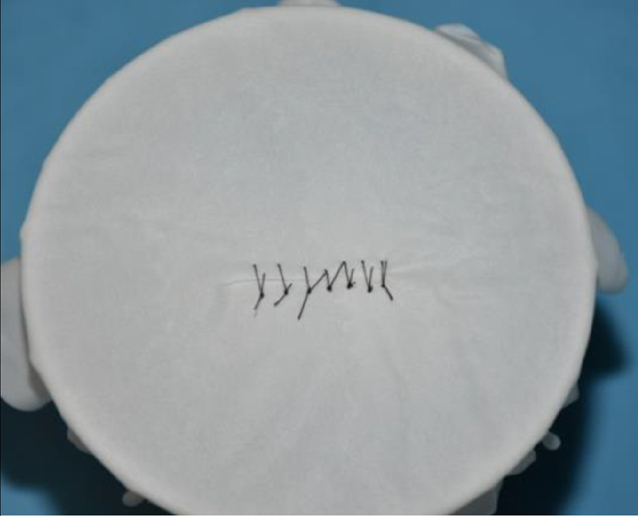
Exercise on silicon tube	1 h	Silicon tube, nylon suture thread 8-0, scissors, curved microsurgical needle holder, microsurgical forceps	Cut the silicone tube obtained from a butterfly fleece Then place the suture on the anterior wall fist and then on the posterior one.	Learn basic suturing technique (vessel suture model)	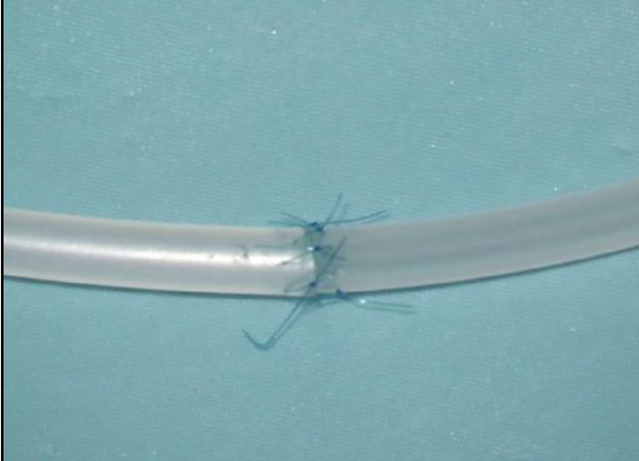
Suture on rubber mouse	1 h	Rubber mouse, nylon suture thread 8-0, scissors, curved microsurgical needle holder, microsurgical forceps	Placing the rubber mouse on the operating field, insert the rubber tube (green) between two spikes inside the rubber mouse. Cut the rubber tube and place the suture	Learn basic suturing technique (vessel suture model)	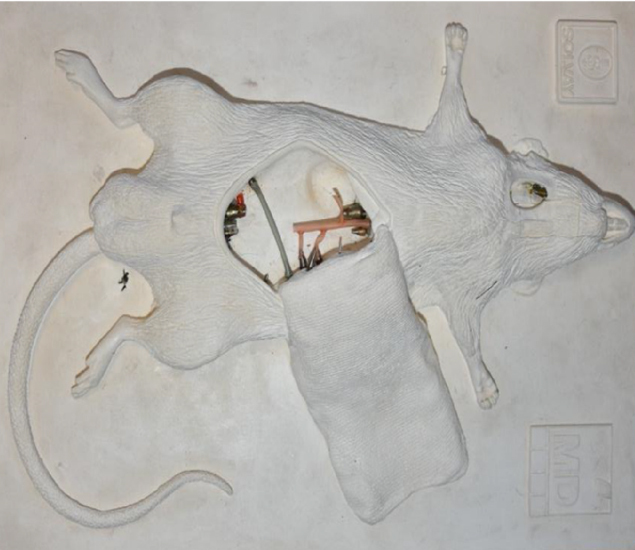
Suture on noodles	1 h	Precooked Japanese noodles, patches, nylon suture thread 10-0, scissors, curved microsurgical needle holder, microsurgical forceps	Rehydrate the noodles with boiling water. Take a noodle and fixe it with two patches at the ends. Cut the noodle and suture it. Using a 10-0 suture thread facilitates the exercise, as a larger suture calibre could destroy the noodle.	Learn basic suturing technique (nerve suture model)	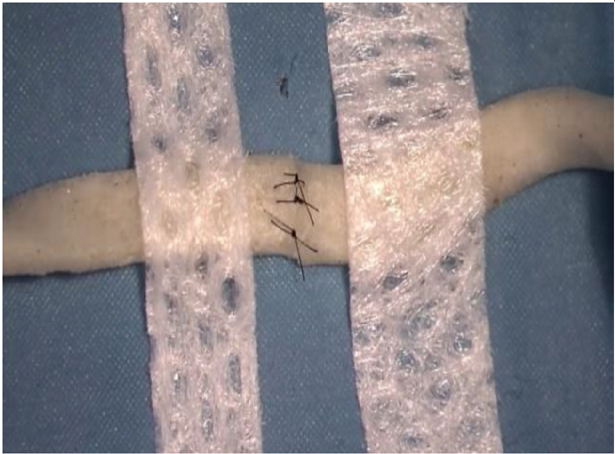
Chicken thigh	18 h	Chicken thigh, colored background, microsurgical needle holder, scissors and forceps, vascular approximator, 8–0 or 9–0 nylon suture	Dissect the tissue to identify femoral vessels. Prepare the operative field by placing the background and the vascular approximator. Perform adventectomy and cut the vessel. Start the anastomosis from anterior or posterior wall.	Training on tissue dissection, vascular anastomosis and neurorrhaphy	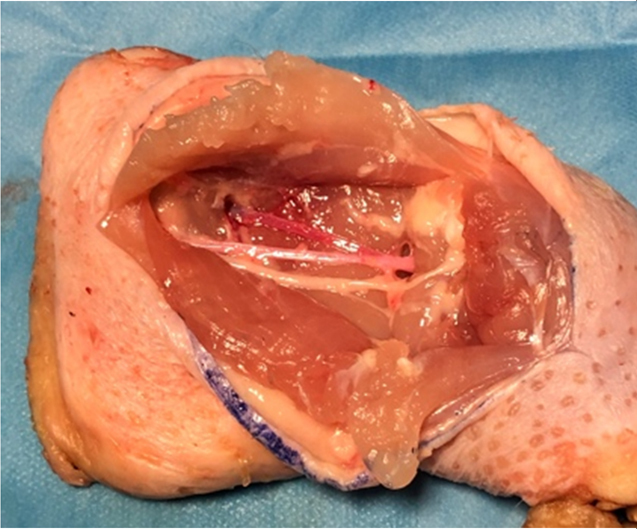

Three exercises (1 h each) were aimed at developing fine manual skills and dexterity. They included working with sushi chopsticks, making the letters embroidered on gauze and training with eyelet needles [24], 25] (Video 1).

**Video 1:**
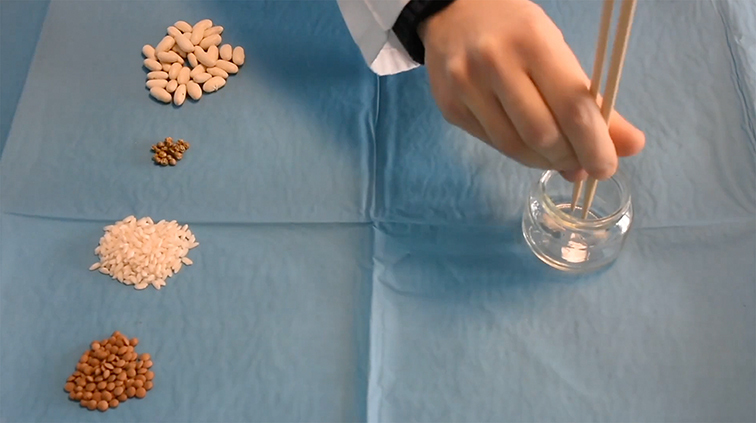
Exercises with sushi chopsticks, on gauze and with eyelet needles.

The exercise (1 h) executed on the grape berry was meant to improve manual finesse and tissue dissection [26] (Video 2).

**Video 2:**
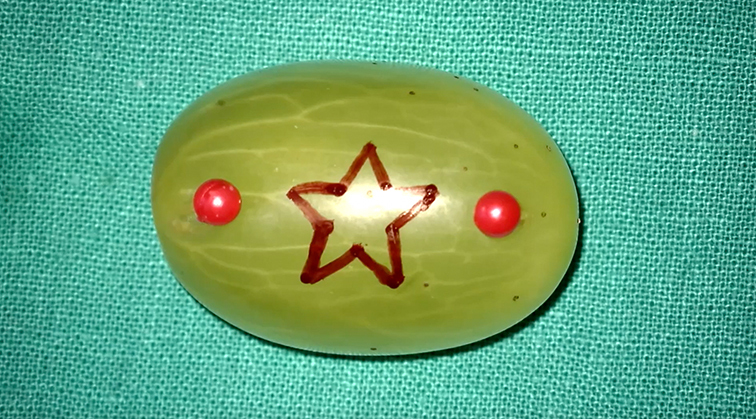
Exercise on the grape berry.

Three exercises (1 h each) were aimed at learning the basic suturing technique. They included exercises on the plate, on the silicone tube or the rubber mouse (vascular suture models) [12], 27] (Video 3) and the exercise on noodles (nerve suture model) [3] (Video 4). The last 18 h of the program were designed with training on the chicken thigh model (Videos 5 and 6). This model was chosen for training on tissue dissection, vascular anastomosis and neurorrhaphy on tissue similar to the living ones [8], 10].

**Video 3:**
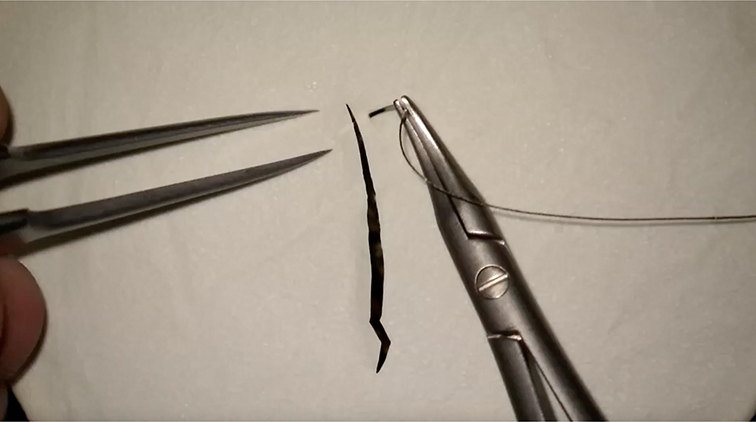
Exercises on the plate, on the silicone tube or on the rubber mouse (vascular suture models).

**Video 4:**
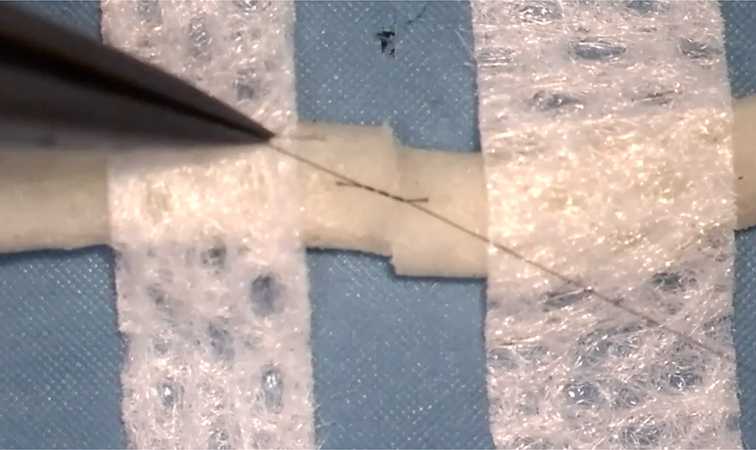
Exercise on noodles (nerve suture model).

**Video 5:**
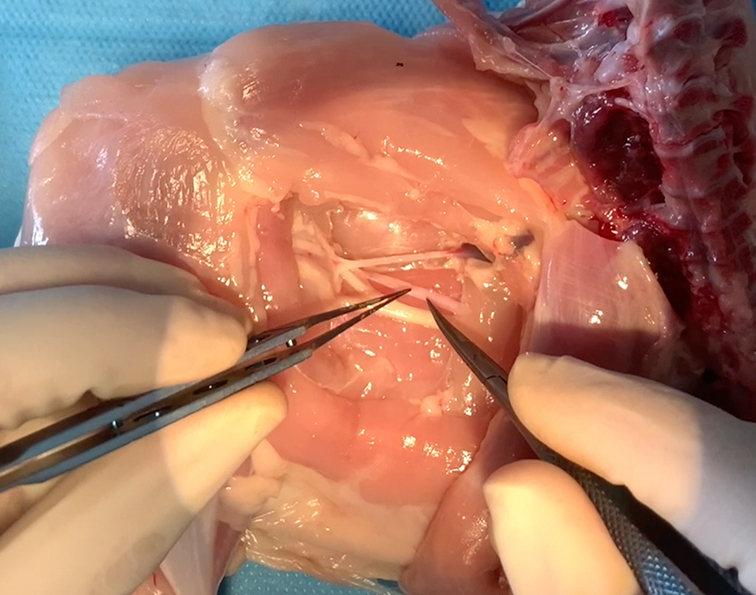
Exercise on the chicken thigh (pt1).

**Video 6:**
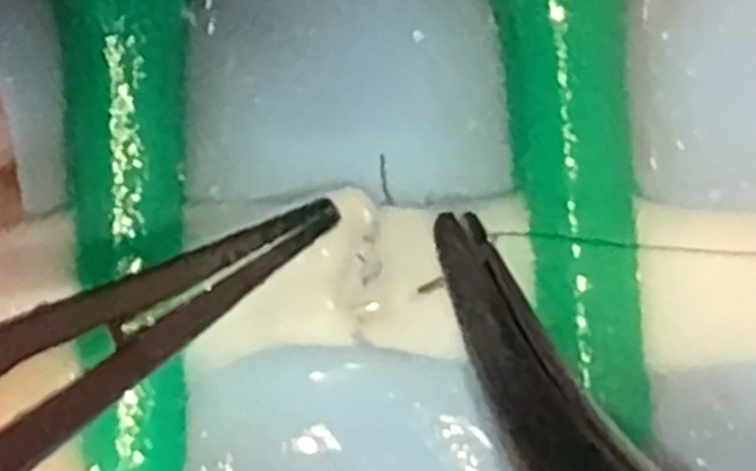
Exercise on the chicken thigh (pt2).

## Results

All trainees were examined before and after the proposed program by 3 senior microsurgeons through the “Queen Mary University London microsurgery global rating scale” parameters. Residents were requested to perform a posterior-wall-first technique end-to-end microvascular anastomosis on the chicken femoral artery within 20 min.

**Figure 1:**
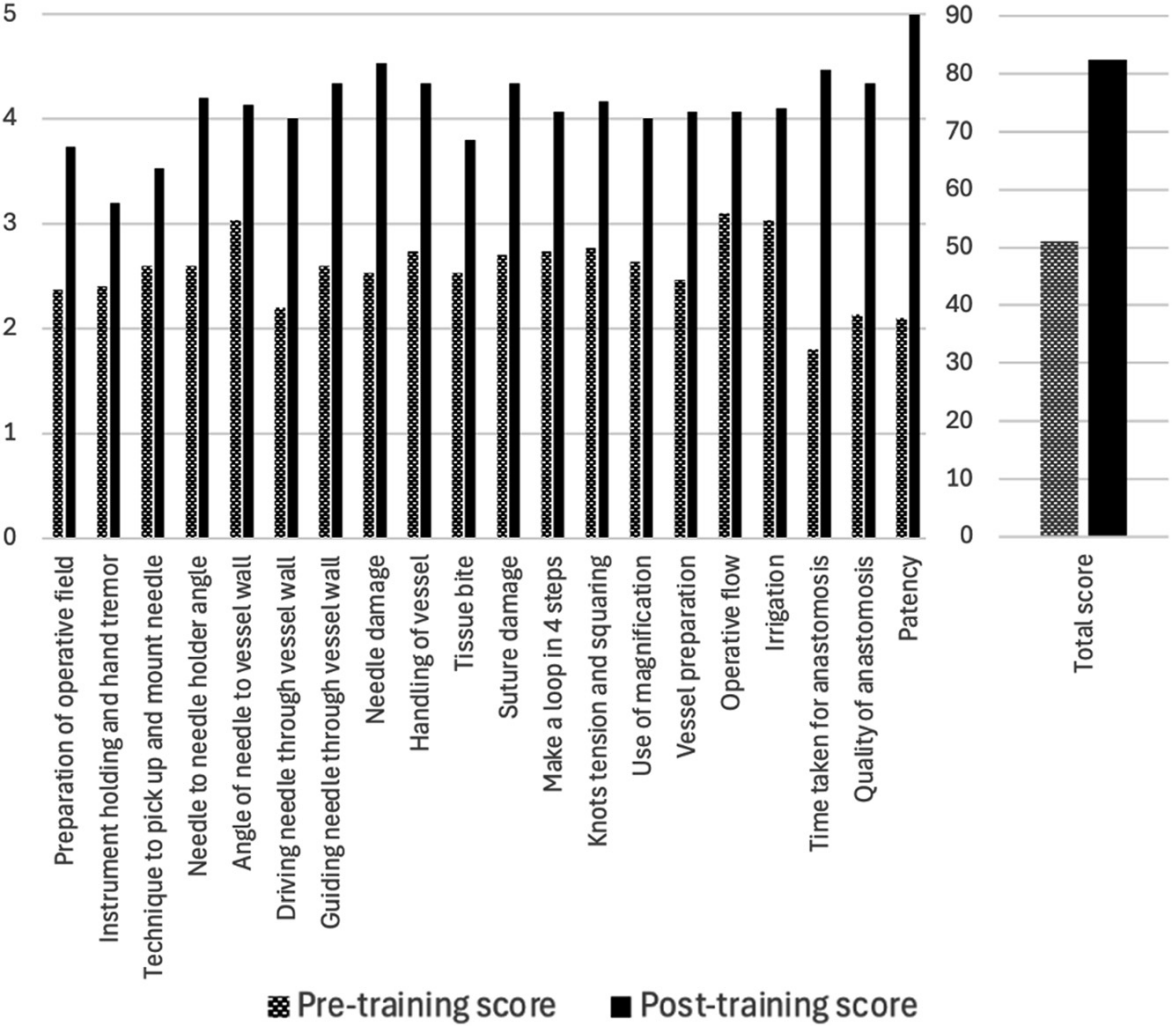
Histograms showing pre- and post-training scores.

The statistical analysis of pre and post training scores revealed a statistically significant improvement in each item of the “Queen Mary University London microsurgery global rating scale” (Figure 1). Moreover, while the total lowest score of pre training was 28 (range 28–72), at the end of the program it was 65 (range 65–98). The results are summarized in Table 2.

**Table 2:** Statistical analysis comparing pre- and post-training scores among the 30 trainees of the program.

Variable (n=30) (mean, range)	Pre-training score	Post-training score	Mean difference	95 % CI	p-Value
Preparation and organization of operative field	2.37 (0–3)	3.73 (1–5)	1.37	0.9451–1.7883	**<**0.001
Instrument holding and hand tremor	2.40 (1–5)	3.20 (1–5)	0.80	0.4279–1.1721	**<**0.001
Technique to pick up and mount needle	2.60 (0–5)	3.53 (1–5)	0.93	0.4741–1.3926	**<**0.001
Needle to needle holder angle	2.60 (0–5)	4.20 (3–5)	1.60	1.1549–2.0451	<0.001
Angle of needle to vessel wall	3.03 (0–5)	4.13 (1–5)	1.10	0.7035–1.4965	**<**0.001
Driving needle through vessel wall	2.20 (0–5)	4.00 (1–5)	1.80	1.2774–2.3226	<0.001
Guiding needle through vessel wall	2.60 (0–5)	4.33 (1–5)	1.73	1.1792–2.2875	<0.001
Needle damage	2.53 (0–5)	4.53 (1–5)	2.00	1.3277–2.6723	<0.001
Handling of vessel	2.73 (0–5)	4.33 (1–5)	1.60	0.9908–2.2092	<0.001
Tissue bite	2.53 (1–5)	3.80 (1–5)	1.27	0.7302–1.8032	<0.001
Suture damage	2.70 (0–5)	4.33 (1–5)	1.63	1.0172–2.2495	<0.001
Make a loop in 4 steps “pick, loop, pick, tighten”	2.73 (1–5)	4.07 (1–5)	1.33	0.8398–1.8269	<0.001
Knots tension and squaring	2.77 (0–5)	4.17 (0–5)	1.40	0.8848–1.9152	<0.001
Use of magnification	2.63 (0–5)	4.00 (1–5)	1.37	0.7744–1.959	<0.001
Vessel preparation for anastomosis	2.47 (1–5)	4.07 (1–5)	1.60	1.1038–2.0962	<0.001
Operative flow	3.10 (0–5)	4.07 (3–5)	0.97	0.462–1.4713	<0.001
Irrigation	3.03 (0–5)	4.10 (0–5)	1.07	0.64–1.4934	<0.001
Time taken for anastomosis (up to 10 stitches)	1.80 (0–3)	4.47 (1–5)	2.67	2.1635–3.1699	<0.001
Quality of anastomosis	2.13 (0–5)	4.33 (3–5)	2.20	1.6084–2.7916	<0.001
Patency	2.10 (0–5)	5.00 (5–5)	2.90	1.9783–3.8217	<0.001
Total score	51.07 (28–72)	82.40	31.33	26.9794–35.6872	<0.001

All trainees were able to perform the anastomosis and the fields in which major mistakes were made were knots tension and squaring, irrigation and time taken for anastomosis, 5/30 trainees being unable to complete the anastomosis within 20 min. The comparison of each score of the “Queen Mary University London microsurgery global rating scale” during pre- and post-training is shown in Figure 1.

## Discussion

Microsurgical training is fundamental for Plastic Surgery residents’ surgical education, preparing them to apply microsurgical techniques in clinical practice. Microsurgery requires constant and dedicated preclinical training that is divided into steps of increasing difficulty: first on non-animal models, then on non-living animal models and finally on animal models [28]. Usually, microsurgical training on animal models, especially rats, better replicates the clinical scenario in humans, but requires dedicated laboratories and personnel, funding for animals feeding and care, to follow severe ethical rules and finally involves animals’ sacrifice. In line with the growing interest in animal rights and 3R principles (refine, replace and reduce) [2], alternatives for microsurgical training on non-living models are desirable, reserving the use of live animals only for the more advanced phases of training.

The proposed program is easily accessible and applicable and consists of a variety of cost-effective exercises, simple to replicate, with easy to find materials. Most basic programs are designed only with the first phases on chicken thigh models or non-living models and the last phase on living rats. In our proposal, the variety of exercises allows more practice on non-living models working on all the different skills required for microsurgery. It was built by choosing heterogeneous exercises, also proposing alternatives for the same skill to be practiced, to make the training more stimulating and less monotonous for the trainee. The chosen exercises allow the trainees to develop and improve basic abilities:

– Practicing with sushi chopsticks, gauze and eyelet needles permits to build up fine manual abilities and dexterity;
– Grape berry dissection enables to increase the fine dissection and the manual finesse under magnification;
– Training on silicon tube, rubber mouse and noodles afford to learn basic suturing techniques for vascular and nervous patterns;
– Exercising on chicken thigh allows to improve tissue dissection technique, vessel harvesting for anastomosis and vascular and nerve sewing techniques.

Although the first macro exercises may seem useless, they are fundamental for the trainee to understand the right hand and wrist movements, to familiarize with microsurgical instruments and treads and to practice hand steadiness.

Most of the program involves exercises on the chicken thigh model (18 h), as it empowers several abilities such as tissue dissection, fine neuro-vascular bundle manipulation, vessel isolation and adventectomy for anastomosis, to practice how to prepare the surgical field (to place the background, to pose the vascular clamps and to irrigate), and to train all suture techniques on vessels or nerves.

A 25-h setup program is easily organized, however, giving trainees the basic microsurgical skills needed to start a supervised microsurgical practice.

All residents were included in the study without any selection based on previous microsurgical background, since our study purpose was not to assess post-training improvement based on specific experience levels, but to evaluate the overall feasibility and ability to enhance the progress of the proposed program.

Our results show that each trainee was able to make a well-made end-to-end anastomosis on the chicken femoral artery at the end of the proposed program. The most frequent failures were about anastomosis speed/time of execution, the preparation of operative field and knots squaring, which can be all improved with more practice.

Our results suggest that the proposed training, even if it consists of simple exercises, could help to improve and maintain the microsurgical techniques already acquired.

Certainly, exercises on non-living models, despite they offer a good model for microsurgical training, make training easier and less realistic compared with ones on living models. One of the primary drawbacks of non-living models is their inability to replicate biological reactions such as bleeding, tissue healing, and physiological changes. In real microsurgery, factors like vessel elasticity, blood flow, and tissue response to suturing play a critical role, which non-living models fail to simulate. Non-living models do not allow for blood flow assessment, making it difficult to evaluate the patency of microvascular sutures or to practice of troubleshooting techniques for complications like thrombosis or vasospasm. Therefore, they do not fully replicate the challenges faced in live surgery. Factors such as time pressure, patient variability, unexpected complications and the need of real-time decision-making are absent, which may create a gap in skill transferability to clinical practice. The absence of real-life stressors may have an impact on the residents’ ability to adapt to actual surgical environments. Without exposure to these stressors during training, residents may struggle to develop adaptability and self-confidence when shifting to real-life procedures. The inclusion of stress-inducing elements, such as time constraints or simulated complications, into training programs could help to bridge this gap.

Despite their limitations, non-living models allow the trainee to practice microsurgery from the beginning and to become familiar with the microscope, microsurgical instruments, and treads, to learn how to handle microsurgical needles and put well-squared knots, to improve dissection techniques, especially on non-living animal models, to learn how to perform adventectomy and cut vessels stumps preparing them for anastomosis and to practice both vascular and nerve suture.

Additionally, non-living model practice can be supplemented with high-fidelity simulations and expedients that may better prepare residents for the demands of real microsurgical procedures or advanced training on living animals.

For example, there are several methods to simulate the pumping flow, such as the injection of colored fluid into the vessel [14], 29], 30] and the use of a peristaltic pump commercially available [31]. These tools can find application in intermediate level training. Moreover, direct vision under magnification of the lumen of the anastomosis opened longitudinally [8] can be useful to overcome the difficulty of valuing the patency of the anastomosis. There are many microsurgical courses worldwide that differ in difficulty, type of models used (non-living vs. living or both) and usually last 1 or 2 weeks. It is established that an intensive and focused microsurgical workout, through step-by-step increasingly difficult exercises, allows the trainee to gain a microsurgical technique almost comparable to that of experienced microsurgeons, due to the importance of repeating the same surgical acts in a relatively short time [32].

A program based on non-living models, regardless of all concerns about it does not use the optimal simulation reality models, allows the development of the needed basic microsurgical skills, to perform and repeat the fundamental surgical gestures in the execution of vascular anastomoses, thus can prepare the trainee for the most advanced phases of training.

Despite non-living models have different drawbacks and will probably never be able to totally replace living models, they consent to practice microsurgery anytime and almost anywhere with only the use of an operative microscope and microsurgical instruments and give the trainee with the essential skills and model knowledge to further train independently. This study was designed to provide a cheap, easy organizable and standardized basic program on non-living models in which we propose a wide variety of exercises of increasing difficulty that make training stimulating and challenging, even on non-living models.

The main limitation of this study is the small number of trainees to which it has been applied, being 30 participants not a significant sample. Another limitation is that we did not compare the results of non-living models training with the ones on living models. However, the training program proposed is a valuable reference for other training programs or surgical residents themselves.

## Conclusions

The described program represents a basic microsurgical course entirely based on non-living models, standardized following the guidelines of the Italian Society for Microsurgery (SIM) that proposes a step-by-step guided training that was previously missing in the current literature. It gives trainees with the basic microsurgical skills needed to move on to rat *in vivo* training or supervised clinical practice. This program makes microsurgical training easily accessible, stimulating and organizable and could help to spread microsurgical practice among residents or young surgeons. However, the reiteration of the practical test among trainees in order to evaluate the long-term retention of acquired skills, the supplementation of high-fidelity simulations and the enrollment of a larger sample in a multi-center study would be beneficial to assess the validity of the proposed program.
